# Supplementary material for: Evolution of alternative biosynthetic pathways for vitamin C following plastid acquisition in photosynthetic eukaryotes
Source: eLife. 2015 Mar 13;4:e06369. doi: 10.7554/eLife.06369 (PMC4396506; doi:10.7554/eLife.06369)
Supplement: Supplementary file 1. — Distribution of GULO and GLDH in opisthokont and apusomonad genomes. Genomes of the opisthokonts (including animals and fungi) were examined for the presence of GULO and GLDH. Strikethrough indicates non-functional pseudogenes. The absence of GULO is well documented in the known ascorbate auxotrophs such as haplorhine primates, guinea pigs, bats, teleost fish and passerine birds. Some fungi (e.g., ascomycetes) use D-arabinonolactone oxidase to produce five carbon ascorbate analogue, erythroascorbate. D-arabinonolactone oxidase has different substrate specificity to GULO, but exhibits a high degree of sequence similarity and has been classed as GULO in the table. DOI: http://dx.doi.org/10.7554/eLife.06369.015 [file elife06369s001.docx]

|  |  | Group |  | *Species* |  |  |
| --- | --- | --- | --- | --- | --- | --- |
| Metazoa | Deuterostomes | vertebrate | Rodents | *Mus musculus* | GULO |  |
|  |  |  |  | *Rattus norvegicus* | GULO |  |
|  |  |  |  | *Cavia porcellus* | ~~GULO~~ |  |
|  |  |  |  | *Orcinus orca* | GULO |  |
|  |  |  | Primates | *Otolemur garnettii* | GULO |  |
|  |  |  |  | *Gorilla gorilla* | ~~GULO~~ |  |
|  |  |  |  | *Homo sapiens* | ~~GULO~~ |  |
|  |  |  | bats | *Rousettus leschenaultii* |  |  |
|  |  |  |  | *Pteropus vampyrus* |  |  |
|  |  |  | Reptile | *Alligator sinensis* | GULO |  |
|  |  |  | Birds | *Falco peregrinus* | GULO |  |
|  |  |  |  | *Gallus gallus* | GULO |  |
|  |  |  |  | *Zonotrichia albicollis* |  |  |
|  |  |  | Amphibian | *Xenopus laevis* | GULO |  |
|  |  |  | Fish | *Danio rerio* |  |  |
|  |  |  |  | *Takifugu rubripes* |  |  |
|  |  |  |  | *Scyliorhinus torazame* | GULO |  |
|  |  |  |  | *Triakis scyllium* | GULO |  |
|  |  | cephalochordata |  | *Branchiostoma floridae* | GULO |  |
|  |  | urochordata |  | *Ciona intestinalis* | GULO |  |
|  |  | hemichordates |  | *Saccoglossus kowalevskii* |  |  |
|  |  | echinoderms |  | *Strongylocentrotus purpuratus* | GULO |  |
|  | Lophotrochozoans | platyhelminthes |  | *Schistosoma mansoni* |  |  |
|  |  | annelids |  | *Capitella teleta* | GULO |  |
|  |  |  |  | *Helobdella robusta* |  |  |
|  |  | molluscs |  | *Aplysia californica* | GULO |  |
|  |  |  |  | *Haliotis discus hannai* | GULO |  |
|  |  |  |  | *Lottia gigantea* | GULO |  |
|  |  |  |  | *Crassostrea gigas* |  |  |
|  | Ecdysozoans | nematodes |  | *Caenorhabditis elegans* |  |  |
|  |  |  |  | *Brugia malayi* |  |  |
|  |  |  |  | *Caenorhabditis brenneri* |  |  |
|  |  |  |  | *Caenorhabditis briggsae* |  |  |
|  |  |  |  | *Caenorhabditis japonica* |  |  |
|  |  |  |  | *Caenorhabditis remanei* |  |  |
|  |  |  |  | *Loa loa* |  |  |
|  |  |  |  | *Pristionchus pacificus* |  |  |
|  |  |  |  | *Trichinella spiralis* |  |  |
|  |  | arthropods | Chelicerata | *Ixodes scapularis* | GULO |  |
|  |  |  |  | *Tetranychus urticae* |  |  |
|  |  |  | Myriapoda | *Strigamia maritima* | GULO |  |
|  |  |  | Crustacea | *Daphnia pulex* |  |  |
|  |  |  | Insecta | *Tribolium castaneum* |  |  |
|  |  |  |  | *Aedes aegypti* |  |  |
|  |  |  |  | *Anopheles darlingi* |  |  |
|  |  |  |  | *Anopheles gambiae* |  |  |
|  |  |  |  | *Culex quinquefasciatus* |  |  |
|  |  |  |  | *Drosophila ananassae* |  |  |
|  |  |  |  | *Drosophila erecta* |  |  |
|  |  |  |  | *Drosophila grimshawi* |  |  |
|  |  |  |  | *Drosophila melanogaster* |  |  |
|  |  |  |  | *Drosophila mojavensis* |  |  |
|  |  |  |  | *Drosophila persimilis* |  |  |
|  |  |  |  | *Drosophila pseudoobscura* |  |  |
|  |  |  |  | *Drosophila sechellia* |  |  |
|  |  |  |  | *Drosophila simulans* |  |  |
|  |  |  |  | *Drosophila virilis* |  |  |
|  |  |  |  | *Drosophila willistoni* |  |  |
|  |  |  |  | *Drosophila yakuba* |  |  |
|  |  |  |  | *Megaselia scalaris* |  |  |
|  |  |  |  | *Acyrthosiphon pisum* |  |  |
|  |  |  |  | *Rhodnius prolixus* |  |  |
|  |  |  |  | *Apis florea* |  |  |
|  |  |  |  | *Bombus impatiens* |  |  |
|  |  |  |  | *Apis mellifera* |  |  |
|  |  |  |  | *Atta cephalotes* |  |  |
|  |  |  |  | *Nasonia vitripennis* |  |  |
|  |  |  |  | *Bombyx mori* |  |  |
|  |  |  |  | *Danaus plexippus* |  |  |
|  |  |  |  | *Heliconius melpomene* |  |  |
|  |  |  |  | *Pediculus humanus* |  |  |
|  | cnidarians |  |  | *Nematostella vectensis* | GULO |  |
|  | poriferans |  |  | *Amphimedon queenslandica* | GULO |  |
|  | placozoa |  |  | *Trichoplax adhaerens* | GULO |  |
| Choanoflagellata |  |  |  | *Monosiga brevicolis* |  | GLDH |
|  |  |  |  | *Salpingoeca rosetta* |  | GLDH |
| Filasterea |  |  |  | *Capsaspora owczarzaki* | GULO |  |
| Ichtyosporea |  |  |  | *Sphaeroforma artica* |  |  |
|  |  |  |  |  |  |  |
| Fungi | Cryptomycota |  |  | *Rozella allomycis CSF55* |  |  |
|  | Microsporidia |  |  | *Antonospora locustae* |  |  |
|  |  |  |  | *Encephalitozoon cuniculi* |  |  |
|  |  |  |  | *Nematocida parisii* |  |  |
|  | Neocallimastigomycota | |  | *Piromyces sp. E2* |  |  |
|  | Chytridiomycota |  |  | *Batrachochytrium dendrobatidis* |  |  |
|  |  |  |  | *Gonapodya prolifera* |  | GLDH |
|  | Blastocladiomycota |  |  | *Catenaria anguillulae* | GULO |  |
|  | Kickxellomycotina |  |  | *Coemansia reversa* | GULO |  |
|  | Mucoromycotina |  |  | *Mucor circinelloides* | GULO |  |
|  |  |  |  | *Rhizopus oryzae* | GULO |  |
|  | Basidiomycetes |  |  | *Cryptococcus neoformans* | GULO |  |
|  |  |  |  | *Ustilago maydis* | GULO |  |
|  |  |  |  | *Agaricus bisporus* | GULO |  |
|  |  |  |  | *Scleroderma citrinum* | GULO |  |
|  |  |  |  | *Laccaria bicolor* | GULO |  |
|  |  |  |  | *Exidia glandulosa* | GULO |  |
|  |  |  |  | *Postia placenta* | GULO |  |
|  |  |  |  | *Tremella mesenterica* | GULO |  |
|  |  |  |  | *Gloephyllum trabeum* | GULO |  |
|  |  |  |  | *Coniophora puteana* | GULO |  |
|  | Ascomycetes |  |  | *Saccharomyces cerevisiae* | GULO |  |
|  |  |  |  | *Aspergillus niger* | GULO |  |
|  |  |  |  | *Neurospora crassa* | GULO |  |
| Nucleariidae |  |  |  | *Fonticula alba* |  |  |
|  |  |  |  |  |  |  |
| Apusumondida |  |  |  | *Thecamonas trahens* | GULO |  |

**Supplementary File 1: Distribution of *GULO* and *GLDH* in opisthokont and apusomonad genomes**
