# Supplementary material for: Evolution of alternative biosynthetic pathways for vitamin C following plastid acquisition in photosynthetic eukaryotes
Source: eLife. 2015 Mar 13;4:e06369. doi: 10.7554/eLife.06369 (PMC4396506; doi:10.7554/eLife.06369)
Supplement: Supplementary file 2. — Identification of GULO and GLDH in marine microbial eukaryote transcriptomes. Data from the Marine Microbial Eukaryote Transcriptome Sequencing Project (MMETSP, http://marinemicroeukaryotes.org/) were analysed for the terminal enzymes in ascorbate biosynthesis. This dataset contains 679 transcriptomes from 320 different species. Sequence similarity searches used a stringent length cut off to avoid ambiguous results from incompletely sequenced gene products (minimum length 300 amino acids). Using these criteria, we identified GULO or GLDH in 165 species. Although the absence of a gene in a transcriptome cannot be used to infer absence, we found no examples of organisms that possess both GULO and GLDH, even when a more relaxed length criterion was used (minimum length 100 amino acids). Note that the underlined GLDH sequences from the ciliates Myrionecta and Strombidinopsis are 100% identical to sequences recovered from their prey (respectively Geminigera cryophila and Isochrysis galbana). These sequences may therefore be due to contamination. Alternatively, as both these ciliates exhibit kleptoplasty, the presence of algal GLDH sequences in the ciliate transcriptome may also represent examples of plastid-related nuclear genes that are retained and transcribed to aid plastid function (Johnson et al., 2007). In the latter scenario, these ciliates could therefore use GLDH to temporarily synthesise ascorbate during plastid acquisition. DOI: http://dx.doi.org/10.7554/eLife.06369.016 [file elife06369s002.docx]

| **MMETSP ID** | **Kingdom** | **Phlyum** |  | **Genus** | **species** | **strain** | | **GULO** | | **GLDH** | **CAMPEP_ID** | |
| --- | --- | --- | --- | --- | --- | --- | --- | --- | --- | --- | --- | --- |
|  |  |  |  |  |  |  | |  | |  |  | |
| MMETSP0052 | Archaeplastida | Chlorophyta | Chlorophyceae | Polytomella | parva | SAG 63-3 | |  | | GLDH | CAMPEP_0175064752 | |
| MMETSP1180 |  | Chlorophyta | Chlorophyceae | Chlamydomonas | sp cf | CCMP681 | |  | | GLDH | CAMPEP_0119109808 | |
| MMETSP1106 |  | Chlorophyta | Prasinophyceae | Mantoniella | antarctica | SL-175 | |  | | GLDH | CAMPEP_0181362302 | |
| MMETSP1327 |  | Chlorophyta | Prasinophyceae | Micromonas | pusilla | RCC2306 | |  | | GLDH | CAMPEP_0119223812 | |
| MMETSP0804 |  | Chlorophyta | Chlorodendrophyceae | Tetraselmis | astigmatica | CCMP880 | |  | | GLDH | CAMPEP_0117687368 | |
| MMETSP0491 |  | Chlorophyta | Chlorodendrophyceae | Tetraselmis | chuii | PLY429 | |  | | GLDH | CAMPEP_0177757908 | |
| MMETSP0419 |  | Chlorophyta | Chlorodendrophyceae | Tetraselmis | sp. | GSL018 | |  | | GLDH | CAMPEP_0177591958 | |
| MMETSP0817 |  | Chlorophyta | Chlorodendrophyceae | Tetraselmis | striata | LANL1001 | |  | | GLDH | CAMPEP_0183839196 | |
| MMETSP0058 |  | Chlorophyta | Prasinophyceae | Pyramimonas | parkeae | CCMP726 | |  | | GLDH | CAMPEP_0114254942 | |
| MMETSP0033 |  | Chlorophyta | Prasinophyceae | Dolichomastix | tenuilepis | CCMP3274 | |  | | GLDH | CAMPEP_0170132808 | |
| MMETSP1080 |  | Chlorophyta | Prasinophyceae | Micromonas | sp | CCMP1646 | |  | | GLDH | CAMPEP_0181147462 | |
| MMETSP0802 |  | Chlorophyta | Prasinophyceae | Micromonas | sp. | CCMP2099 | |  | | GLDH | CAMPEP_0117629166 | |
| MMETSP0929 |  | Chlorophyta | Prasinophyceae | Ostreococcus | mediterraneus | BCC35000 | |  | | GLDH | CAMPEP_0174584534 | |
| MMETSP0930 |  | Chlorophyta | Prasinophyceae | Ostreococcus | mediterraneus | BCC44000 | |  | | GLDH | CAMPEP_0179604014 | |
| MMETSP0932 |  | Chlorophyta | Prasinophyceae | Ostreococcus | mediterraneus | BCC98000 | |  | | GLDH | CAMPEP_0179621732 | |
| MMETSP0933 |  | Chlorophyta | Prasinophyceae | Ostreococcus | mediterraneus | BCC99000 | |  | | GLDH | CAMPEP_0179647186 | |
| MMETSP0935 |  | Chlorophyta | Prasinophyceae | Ostreococcus | mediterraneus | BCC103000 | |  | | GLDH | CAMPEP_0179661620 | |
| MMETSP0936 |  | Chlorophyta | Prasinophyceae | Ostreococcus | mediterraneus | BCC109000 | |  | | GLDH | CAMPEP_0179690654 | |
| MMETSP0937 |  | Chlorophyta | Prasinophyceae | Ostreococcus | mediterraneus | BCC115000 | |  | | GLDH | CAMPEP_0179708284 | |
| MMETSP0938 |  | Chlorophyta | Prasinophyceae | Ostreococcus | mediterraneus | BCC116000 | |  | | GLDH | CAMPEP_0179723532 | |
| MMETSP0939 |  | Chlorophyta | Prasinophyceae | Ostreococcus | mediterraneus | BCC118000 | |  | | GLDH | CAMPEP_0179735702 | |
| MMETSP0034 |  | Chlorophyta | Prasinophyceae | Nephroselmis | pyriformis | CCMP717 | |  | | GLDH | CAMPEP_0182852242 | |
| MMETSP1316 |  | Chlorophyta | Prasinophyceae | Pycnococcus | provasolii | RCC2336 | |  | | GLDH | CAMPEP_0119193948 | |
| MMETSP1169 |  | Chlorophyta | Prasinophyceae | Pyramimonas | obovata | CCMP722 | |  | | GLDH | CAMPEP_0118920740 | |
| MMETSP0803 |  | Chlorophyta | Prasinophyceae | Crustomastix | stigmata | CCMP3273 | |  | | GLDH | CAMPEP_0183815946 | |
| MMETSP0807 |  | Chlorophyta | Unknown | Picocystis | salinarum | CCMP1897 | |  | | GLDH | CAMPEP_0183832396 | |
| MMETSP1353 | Archaeplastida | Rhodophyta | Porphyridiophyceae | Erythrolobus | australicus | CCMP3124 | |  | | GLDH | CAMPEP_0185837082 | |
| MMETSP1172 |  | Rhodophyta | Porphyridiophyceae | Timspurckia | oligopyrenoides | CCMP3278 | |  | | GLDH | CAMPEP_0182450390 | |
| MMETSP0312 |  | Rhodophyta | Compsopogonophyceae | Compsopogon | coeruleus | SAG 36.94 | |  | | GLDH | CAMPEP_0184690532 | |
| MMETSP0313 |  | Rhodophyta | Porphyridiophyceae | Porphyridium | aerugineum | SAG 1380-2 | |  | | GLDH | CAMPEP_0184699824 | |
| MMETSP0167 |  | Rhodophyta | Rhodellophyceae | Rhodella | maculata | CCMP736 | |  | | GLDH | CAMPEP_0174906552 | |
| MMETSP0314 |  | Rhodophyta | Rhodellophyceae | Rhodella | maculata | CCMP736 | |  | | GLDH | CAMPEP_0184727162 | |
| MMETSP0011 |  | Rhodophyta | Rhodellophyceae | Rhodosorus | marinus | CCMP 769 | |  | | GLDH | CAMPEP_0113961234 | |
| MMETSP0315 |  | Rhodophyta | Rhodellophyceae | Rhodosorus | marinus | UTEX LB 2760 | |  | | GLDH | CAMPEP_0184737942 | |
|  |  |  |  |  |  |  | |  | |  |  | |
| MMETSP0724 | SAR - stramenopiles | Ochrophyta | Bacillariophyceae | Amphiprora | sp. | CCMP467 | |  | | GLDH | CAMPEP_0168760430 | |
| MMETSP0733 |  | Ochrophyta | Bacillariophyceae | Fragilariopsis | kerguelensis | L26-C5 | |  | | GLDH | CAMPEP_0170811962 | |
| MMETSP0744 |  | Ochrophyta | Bacillariophyceae | Nitzschia | punctata | CCMP561 | |  | | GLDH | CAMPEP_0178758508 | |
| MMETSP0014 |  | Ochrophyta | Bacillariophyceae | Nitzschia | sp. | RCC80 | |  | | GLDH | CAMPEP_0113486334 | |
| MMETSP0329 |  | Ochrophyta | Bacillariophyceae | Pseudo-nitzschia | arenysensis | B593 | |  | | GLDH | CAMPEP_0116146726 | |
| MMETSP0139 |  | Ochrophyta | Bacillariophyceae | Pseudo-nitzschia | australis | 10249 10 AB | |  | | GLDH | CAMPEP_0168170388 | |
| MMETSP0327 |  | Ochrophyta | Bacillariophyceae | Pseudo-nitzschia | delicatissima | B596 | |  | | GLDH | CAMPEP_0116090274 | |
| MMETSP1060 |  | Ochrophyta | Bacillariophyceae | Pseudo-nitzschia | pungens | cf. cingulata | |  | | GLDH | CAMPEP_0172377542 | |
| MMETSP1061 |  | Ochrophyta | Bacillariophyceae | Pseudo-nitzschia | pungens | cf. pungens | |  | | GLDH | CAMPEP_0172383674 | |
| MMETSP0318 |  | Ochrophyta | Bacillariophyceae | Amphora | coffeaeformis | CCMP127 | |  | | GLDH | CAMPEP_0170700894 | |
| MMETSP1336 |  | Ochrophyta | Bacillariophyceae | Chaetoceros | cf. neogracile | RCC1993 | |  | | GLDH | CAMPEP_0119463674 | |
| MMETSP1176 |  | Ochrophyta | Bacillariophyceae | Synedropsis | recta cf | CCMP1620 | |  | | GLDH | CAMPEP_0119020186 | |
| MMETSP1064 |  | Ochrophyta | Coscinodiscophyceae | Aulacoseira | subarctica | CCAP 1002/5 | |  | | GLDH | CAMPEP_0172420718 | |
| MMETSP0200 |  | Ochrophyta | Coscinodiscophyceae | Chaetoceros | sp. | GSL56 | |  | | GLDH | CAMPEP_0176481880 | |
| MMETSP0696 |  | Ochrophyta | Coscinodiscophyceae | Extubocellulus | spinifer | CCMP396 | |  | | GLDH | CAMPEP_0178537938 | |
| MMETSP1070 |  | Ochrophyta | Coscinodiscophyceae | Minutocellus | polymorphus | NH13 | |  | | GLDH | CAMPEP_0181022046 | |
| MMETSP1322 |  | Ochrophyta | Coscinodiscophyceae | Minutocellus | polymorphus | RCC2270 | |  | | GLDH | CAMPEP_0185828230 | |
| MMETSP1062 |  | Ochrophyta | Coscinodiscophyceae | Ditylum | brightwellii | Pop1 (SS4) | |  | | GLDH | CAMPEP_0180948762 | |
| MMETSP1063 |  | Ochrophyta | Coscinodiscophyceae | Ditylum | brightwellii | Pop2 (SS10) | |  | | GLDH | CAMPEP_0180987192 | |
| MMETSP0816 |  | Ochrophyta | Coscinodiscophyceae | Proboscia | inermis | CCAP1064/1 | |  | | GLDH | CAMPEP_0171322398 | |
| MMETSP0789 |  | Ochrophyta | Coscinodiscophyceae | Rhizosolenia | setigera | CCMP 1694 | |  | | GLDH | CAMPEP_0178954052 | |
| MMETSP1057 |  | Ochrophyta | Coscinodiscophyceae | Cyclotella | meneghiniana | CCMP 338 | |  | | GLDH | CAMPEP_0172273310 | |
| MMETSP1058 |  | Ochrophyta | Coscinodiscophyceae | Detonula | confervacea | CCMP 353 | |  | | GLDH | CAMPEP_0172315460 | |
| MMETSP0013 |  | Ochrophyta | Coscinodiscophyceae | Skeletonema | costatum | RA080513-05 | |  | | GLDH | CAMPEP_0113392494 | |
| MMETSP0319 |  | Ochrophyta | Coscinodiscophyceae | Skeletonema | marinoi | SM1012Hels-07 | |  | | GLDH | CAMPEP_0115911916 | |
| MMETSP0320 |  | Ochrophyta | Coscinodiscophyceae | Skeletonema | marinoi | SM1012Den-03 | |  | | GLDH | CAMPEP_0115962872 | |
| MMETSP0918 |  | Ochrophyta | Coscinodiscophyceae | Skeletonema | marinoi | skelA | |  | | GLDH | CAMPEP_0184920824 | |
| MMETSP1039 |  | Ochrophyta | Coscinodiscophyceae | Skeletonema | marinoi | FE7 | |  | | GLDH | CAMPEP_0180855890 | |
| MMETSP1040 |  | Ochrophyta | Coscinodiscophyceae | Skeletonema | marinoi | FE60 | |  | | GLDH | CAMPEP_0180890906 | |
| MMETSP0737 |  | Ochrophyta | Coscinodiscophyceae | Thalassiosira | miniscula | CCMP1093 | |  | | GLDH | CAMPEP_0183714268 | |
| MMETSP0403 |  | Ochrophyta | Coscinodiscophyceae | Thalassiosira | rotula | CCMP3096 | |  | | GLDH | CAMPEP_0184802732 | |
| MMETSP1059 |  | Ochrophyta | Coscinodiscophyceae | Thalassiosira | sp. | Th. Sp. FW | |  | | GLDH | CAMPEP_0172337252 | |
| MMETSP1071 |  | Ochrophyta | Coscinodiscophyceae | Thalassiosira | sp. | NH16 | |  | | GLDH | CAMPEP_0181079978 | |
| MMETSP0878 |  | Ochrophyta | Coscinodiscophyceae | Thalassiosira | weissflogii | CCMP1336 | |  | | GLDH | CAMPEP_0171332220 | |
| MMETSP0015 |  | Ochrophyta | Coscinodiscophyceae | Odontella | aurita | isolate 1302-5 | |  | | GLDH | CAMPEP_0113561228 | |
| MMETSP0160 |  | Ochrophyta | Coscinodiscophyceae | Odontella | sinensis | 1884 | |  | | GLDH | CAMPEP_0183293006 | |
| MMETSP0713 |  | Ochrophyta | Fragilariophyceae | Asterionellopsis | glacialis |  | |  | | GLDH | CAMPEP_0184882508 | |
| MMETSP0786 |  | Ochrophyta | Fragilariophyceae | Thalassionema | frauenfeldii | CCMP 1798 | |  | | GLDH | CAMPEP_0178895956 | |
| MMETSP1319 | SAR - stramenopiles | Ochrophyta | Bolidophyceae | Bolidomonas | pacifica | RCC208 | |  | | GLDH | CAMPEP_0182488012 | |
| MMETSP1177 |  | Ochrophyta | Chrysophyceae | Ochromonas | sp | CCMP1899 | |  | | GLDH | CAMPEP_0119053538 | |
| MMETSP1103 |  | Ochrophyta | Chrysophyceae | Paraphysomonas | bandaiensis | Caron Lab | |  | | GLDH | CAMPEP_0185040534 | |
| MMETSP0019 |  | Ochrophyta | Chrysophyceae | Dinobryon | sp. | UTEXLB2267 | |  | | GLDH | CAMPEP_0170084414 | |
| MMETSP0005 |  | Ochrophyta | Chrysophyceae | Ochromonas | sp. | CCMP1393 | |  | | GLDH | CAMPEP_0175018602 | |
| MMETSP1105 |  | Ochrophyta | Chrysophyceae | Ochromonas | sp. | BG-1 | |  | | GLDH | CAMPEP_0173144700 | |
| MMETSP1142 |  | Ochrophyta | Chrysophyceae | Undescribed | Undescribed | CCMP2298 | |  | | GLDH | CAMPEP_0173227554 | |
| MMETSP1174 |  | Ochrophyta | Dictyochophyceae | Dictyocha | speculum | CCMP1381 | |  | | GLDH | CAMPEP_0185793444 | |
| MMETSP1324 |  | Ochrophyta | Dictyochophyceae | Florenciella | sp. | RCC1587 | |  | | GLDH | CAMPEP_0182602380 | |
| MMETSP1344 |  | Ochrophyta | Dictyochophyceae | Florenciella | parvula | CCMP2471 | |  | | GLDH | CAMPEP_0119517320 | |
| MMETSP1068 |  | Ochrophyta | Dictyochophyceae | Pseudopedinella | elastica | CCMP716 | |  | | GLDH | CAMPEP_0172586646 | |
| MMETSP0101 |  | Ochrophyta | Dictyochophyceae | Pteridomonas | Pteridomonas | PT | |  | | GLDH | CAMPEP_0114331146 | |
| MMETSP1173 |  | Ochrophyta | Dictyochophyceae | Rhizochromulina | marina cf | CCMP1243 | |  | | GLDH | CAMPEP_0118976096 | |
| MMETSP0914 |  | Ochrophyta | Pelagophyceae | Aureococcus | anophagefferens | CCMP1850 | |  | | GLDH | CAMPEP_0168919368 | |
| MMETSP1166 |  | Ochrophyta | Pelagophyceae | Chrysoreinhardia | sp | CCMP3193 | |  | | GLDH | CAMPEP_0118909844 | |
| MMETSP1329 |  | Ochrophyta | Pelagophyceae | Genus nov. | species nov. | RCC1024 | |  | | GLDH | CAMPEP_0119270028 | |
| MMETSP0974 |  | Ochrophyta | Pelagophyceae | Undescribed | Undescribed | CCMP2097 | |  | | GLDH | CAMPEP_0184116050 | |
| MMETSP1163 |  | Ochrophyta | Pinguiophyceae | Phaeomonas | parva | CCMP2877 | |  | | GLDH | CAMPEP_0118877966 | |
| MMETSP0947 |  | Ochrophyta | Raphidophyceae | Chattonella | subsalsa | CCMP2191 | |  | | GLDH | CAMPEP_0117755356 | |
| MMETSP0416 |  | Ochrophyta | Raphidophyceae | Heterosigma | akashiwo | NB | |  | | GLDH | CAMPEP_0116777886 | |
| MMETSP0942 | SAR - stramenopiles | Bicosoecida | Bicosoecophyceae | Cafeteria | roebergensis | E4-10 | |  | | GLDH | CAMPEP_0117700910 | |
| MMETSP0198 | SAR -stramenopiles | Labyrinthista | Labyrinthulea | Thraustochytrium | sp. | LLF1b | |  | | GLDH | CAMPEP_0184509154 | |
| MMETSP0958 |  | Labyrinthista | Labyrinthulea | Aurantiochytrium | limacinum | ATCCMYA-1381 | |  | | GLDH | CAMPEP_0171491332 | |
| MMETSP0288 | SAR - alveolates | Apicomplexa | Chomerida | Vitrella | brassicaformis | CCMP3155 | | GULO | |  | CAMPEP_0184601628 | |
| MMETSP0290 |  | Apicomplexa | Chomerida | Chromera | velia | CCMP2878 | | GULO | |  | CAMPEP_0184607438 | |
| MMETSP0798 | SAR - alveolates | Ciliophora | Litostomatea | Myrionecta | rubra | CCMP2563 | |  | | **GLDH*** | CAMPEP_0179387252 | |
| MMETSP0126 |  | Ciliophora | Spirotrichea | Strombidinopsis | asuminatum | SPMC142 | |  | | **GLDH*** | CAMPEP_0176359874 | |
| MMETSP0797 | SAR - alveolates | Dinophyta | Dinophyceae | Dinophysis | acuminata | | DAEP01 | |  | GLDH | CAMPEP_0179265132 |  |
| MMETSP0795 |  | Dinophyta | Dinophyceae | Amoebophrya | sp. | | Ameob2 | |  | GLDH | CAMPEP_0178987552 |  |
| MMETSP0766 |  | Dinophyta | Dinophyceae | Gambierdiscus | australes | | CAWD 149 | |  | GLDH | CAMPEP_0171111466 |  |
| MMETSP0790 |  | Dinophyta | Dinophyceae | Alexandrium | catenella | | OF101 | |  | GLDH | CAMPEP_0171194432 |  |
| MMETSP0093 |  | Dinophyta | Dinophyceae | Alexandrium | monilatum | | CCMP3105 | |  | GLDH | CAMPEP_0175187082 |  |
| MMETSP0382 |  | Dinophyta | Dinophyceae | Alexandrium | tamarense | | CCMP1771 | |  | GLDH | CAMPEP_0116284032 |  |
| MMETSP1032 |  | Dinophyta | Dinophyceae | Lingulodinium | polyedrum | | CCMP 1738 | |  | GLDH | CAMPEP_0117944390 |  |
| MMETSP0228 |  | Dinophyta | Dinophyceae | Protoceratium | reticulatum | | CCMP 1889 | |  | GLDH | CAMPEP_0168468780 |  |
| MMETSP0796 |  | Dinophyta | Dinophyceae | Pyrodinium | bahamense | | pbaha01 | |  | GLDH | CAMPEP_0179060648 |  |
| MMETSP0258 |  | Dinophyta | Dinophyceae | Amphidinium | carterae | | CCMP1314 | |  | GLDH | CAMPEP_0176508106 |  |
| MMETSP0689 |  | Dinophyta | Dinophyceae | Amphidinium | massartii | | CS-259 | |  | GLDH | CAMPEP_0178405678 |  |
| MMETSP0784 |  | Dinophyta | Dinophyceae | Gymnodinium | catenatum | | GC744 | |  | GLDH | CAMPEP_0117538690 |  |
| MMETSP0027 |  | Dinophyta | Dinophyceae | Karenia | brevis | | CCMP2229 | |  | GLDH | CAMPEP_0173639946 |  |
| MMETSP0201 |  | Dinophyta | Dinophyceae | Karenia | brevis | | Wilson | |  | GLDH | CAMPEP_0114789702 |  |
| MMETSP0527 |  | Dinophyta | Dinophyceae | Karenia | brevis | | SP3 | |  | GLDH | CAMPEP_0177816518 |  |
| MMETSP0573 |  | Dinophyta | Dinophyceae | Karenia | brevis | | SP1 | |  | GLDH | CAMPEP_0117100536 |  |
| MMETSP1015 |  | Dinophyta | Dinophyceae | Karlodinium | micrum | | CCMP2283 | |  | GLDH | CAMPEP_0169066598 |  |
| MMETSP0224 |  | Dinophyta | Dinophyceae | Togula | jolla | | CCCM 725 | |  | GLDH | CAMPEP_0170568846 |  |
| MMETSP0503 |  | Dinophyta | Dinophyceae | Heterocapsa | rotundata | | SCCAP K-0483 | |  | GLDH | CAMPEP_0168668166 |  |
| MMETSP0116 |  | Dinophyta | Dinophyceae | Durinskia | baltica | | CSIRO CS-38 | |  | GLDH | CAMPEP_0170236138 |  |
| MMETSP0118 |  | Dinophyta | Dinophyceae | Glenodinium | foliaceum | | CCAP 1116/3 | |  | GLDH | CAMPEP_0167882522 |  |
| MMETSP0120 |  | Dinophyta | Dinophyceae | Kryptoperidinium | foliaceum | | CCMP 1326 | |  | GLDH | CAMPEP_0176099136 |  |
| MMETSP0370 |  | Dinophyta | Dinophyceae | Peridinium | aciculiferum | | PAER-2 | |  | GLDH | CAMPEP_0183440480 |  |
| MMETSP0367 |  | Dinophyta | Dinophyceae | Scrippsiella | aff. hangoei | | SHHI-4 | |  | GLDH | CAMPEP_0177245680 |  |
| MMETSP0270 |  | Dinophyta | Dinophyceae | Scrippsiella | trochoidea | | CCMP3099 | |  | GLDH | CAMPEP_0115220328 |  |
| MMETSP0267 |  | Dinophyta | Dinophyceae | Prorocentrum | minium | | CCMP2233 | |  | GLDH | CAMPEP_0176691516 |  |
| MMETSP0227 |  | Dinophyta | Dinophyceae | Polarella | glacialis | | CCMP 1383 | |  | GLDH | CAMPEP_0115097326 |  |
| MMETSP1110 |  | Dinophyta | Dinophyceae | Symbiodinium | sp. | | CCMP421 | |  | GLDH | CAMPEP_0181506954 |  |
| MMETSP1122 |  | Dinophyta | Dinophyceae | Symbiodinium | sp. | | Mp | |  | GLDH | CAMPEP_0169699412 |  |
| MMETSP1036 |  | Dinophyta | Dinophyceae | Azadinium | spinosum | | 3D9 | |  | GLDH | CAMPEP_0180506502 |  |
| MMETSP0109 | SAR - rhizaria | Cercozoa | Chlorarachniophyceae | Chlorarachnion | reptans | | CCCM449 | |  | GLDH | CAMPEP_0114533342 |  |
| MMETSP0110 |  | Cercozoa | Chlorarachniophyceae | Gymnochlora | sp. | | CCMP2014 | |  | GLDH | CAMPEP_0167755114 |  |
| MMETSP0042 |  | Cercozoa | Chlorarachniophyceae | Lotharella | amoebiformis | | CCMP2058 | |  | GLDH | CAMPEP_0118602118 |  |
| MMETSP0041 |  | Cercozoa | Chlorarachniophyceae | Lotharella | globosa | | LEX01 | |  | GLDH | CAMPEP_0114066318 |  |
| MMETSP0111 |  | Cercozoa | Chlorarachniophyceae | Lotharella | globosa | | CCCM811 | |  | GLDH | CAMPEP_0167771192 |  |
| MMETSP0040 |  | Cercozoa | Chlorarachniophyceae | Lotharella | oceanica | | CCMP622 | |  | GLDH | CAMPEP_0170200418 |  |
| MMETSP0113 |  | Cercozoa | Chlorarachniophyceae | Norrisiella | sphaerica | | BC52 | |  | GLDH | CAMPEP_0184481424 |  |
| MMETSP1052 |  | Cercozoa | Chlorarachniophyceae | Bigelowiella | natans | | CCMP623 | |  | GLDH | CAMPEP_0169537218 |  |
| MMETSP1054 |  | Cercozoa | Chlorarachniophyceae | Bigelowiella | natans | | CCMP1259 | |  | GLDH | CAMPEP_0169590640 |  |
| MMETSP1055 |  | Cercozoa | Chlorarachniophyceae | Bigelowiella | natans | | CCMP1258.1 | |  | GLDH | CAMPEP_0172236408 |  |
|  |  |  |  |  |  | |  | |  |  |  |  |
| MMETSP0799 | CCTH | Cryptista | Cryptophyceae | Geminigera | cryophila | | CCMP2564 | |  | GLDH | CAMPEP_0179423788 |  |
| MMETSP1102 |  | Cryptista | Cryptophyceae | Geminigera | sp. | | Caron Lab | |  | GLDH | CAMPEP_0173111424 |  |
| MMETSP0046 |  | Cryptista | Cryptophyceae | Guillardia | theta | | CCMP 2712 | |  | GLDH | CAMPEP_0113795640 |  |
| MMETSP0114 |  | Cryptista | Cryptophyceae | Goniomonas | sp | | m | |  | GLDH | CAMPEP_0114542378 |  |
| MMETSP0043 |  | Cryptista | Cryptophyceae | Hemiselmis | andersenii | | CCMP644 | |  | GLDH | CAMPEP_0114152730 |  |
| MMETSP1041 |  | Cryptista | Cryptophyceae | Hemiselmis | andersenii | | CCMP439 | |  | GLDH | CAMPEP_0172013522 |  |
| MMETSP1042 |  | Cryptista | Cryptophyceae | Hemiselmis | andersenii | | CCMP1180 | |  | GLDH | CAMPEP_0169463190 |  |
| MMETSP1043 |  | Cryptista | Cryptophyceae | Hemiselmis | andersenii | | CCMP441 | |  | GLDH | CAMPEP_0172054608 |  |
| MMETSP1357 |  | Cryptista | Cryptophyceae | Hemiselmis | rufescens | | PCC563 | |  | GLDH | CAMPEP_0173467796 |  |
| MMETSP0986 |  | Cryptista | Cryptophyceae | Undescribed | Undescribed | | CCMP2293 | |  | GLDH | CAMPEP_0180162100 |  |
| MMETSP0780 |  | Cryptista | Palpitia | Palpitomonas | bilix | | NIES-2562 | | GULO |  | CAMPEP_0113872476 |  |
| MMETSP1333 | CCTH | Haptophyta | Prymnesiophyceae | Scyphosphaera | apsteinii | | RCC1455 | |  | GLDH | CAMPEP_0119314704 |  |
| MMETSP0164 |  | Haptophyta | Prymnesiophyceae | Coccolithus | pelagicus | | PLY182g | |  | GLDH | CAMPEP_0183358322 |  |
| MMETSP0595 |  | Haptophyta | Prymnesiophyceae | Isochrysis | galbana | | CCMP1323 | |  | GLDH | CAMPEP_0183615330 |  |
| MMETSP1150 |  | Haptophyta | Prymnesiophyceae | Emiliania | huxleyi | | PLY M219 | |  | GLDH | CAMPEP_0181815204 |  |
| MMETSP1154 |  | Haptophyta | Prymnesiophyceae | Emiliania | huxleyi | | CCMP370 | |  | GLDH | CAMPEP_0182129386 |  |
| MMETSP1363 |  | Haptophyta | Prymnesiophyceae | Gephyrocapsa | oceanica | | RCC1303 | |  | GLDH | CAMPEP_0185313218 |  |
| MMETSP1136 |  | Haptophyta | Prymnesiophyceae | Pleurochrysis | carterae | | CCMP645 | |  | GLDH | CAMPEP_0169947630 |  |
| MMETSP0006 |  | Haptophyta | Prymnesiophyceae | Prymnesium | parvum | | Texoma1 | |  | GLDH | CAMPEP_0182802736 |  |
| MMETSP1094 |  | Haptophyta | Prymnesiophyceae | Chrysochromulina | brevifilum | | UTEX LB 985 | |  | GLDH | CAMPEP_0174694426 |  |
| MMETSP0143 |  | Haptophyta | Prymnesiophyceae | Chrysochromulina | polylepis | | CCMP1757 | |  | GLDH | CAMPEP_0183020038 |  |
| MMETSP0287 |  | Haptophyta | Prymnesiophyceae | Chrysochromulina | rotalis | | UIO044 | |  | GLDH | CAMPEP_0115859226 |  |
|  |  |  |  |  |  | |  | |  |  |  |  |
| MMETSP0417 | Amoebozoa | Lobosa | Dactylopodida | Mayorella | sp | | BSH-02190019 | | GULO |  | CAMPEP_0174236278 |  |
| MMETSP0439 |  | Unknown | Unknown | Stereomyxa | ramosa | | Chinc5 | | GULO |  | CAMPEP_0174271394 |  |
|  |  |  |  |  |  | |  | |  |  |  |  |
| MMETSP0759 | Excavata | Percolozoa | Percolatea | Percolomonas | cosmopolitus | | WS | | GULO |  | CAMPEP_0117443612 |  |
|  |  |  |  |  |  | |  | |  |  |  |  |
| MMETSP0105 | Opisthokonta | Choanoflagellatea | Acanthoecidae | Acanthoeca-like |  | | 10tr | |  | GLDH | CAMPEP_0182922258 |  |
|  |  |  |  |  |  | |  | |  |  |  |  |

**Supplementary File 2: Identification of *GULO* and *GLDH* in marine microbial eukaryote transcriptomes**
