# Supplementary material for: Evolution of alternative biosynthetic pathways for vitamin C following plastid acquisition in photosynthetic eukaryotes
Source: eLife. 2015 Mar 13;4:e06369. doi: 10.7554/eLife.06369 (PMC4396506; doi:10.7554/eLife.06369)
Supplement: Supplementary file 3. — Identification of VTC2 in marine microbial eukaryote transcriptomes. Data from the Marine Microbial Eukaryote Transcriptome Sequencing Project (MMETSP) was analysed for VTC2, encoding GDP-l-galactose phosphorylase, the first committed step in land plant ascorbate biosynthesis. Sequence similarity searches used a stringent cut off to avoid ambiguous results from incompletely sequenced gene products (minimum length 300 amino acids). VTC2 was identified in 37 species, all of which belong to the Chlorophyta. Genes exhibiting weak similarity to GDP-l-galactose phosphorylase, which may represent homologues of GDP-D-glucose phosphorylase (Adler et al., 2011), were not included in these results. DOI: http://dx.doi.org/10.7554/eLife.06369.017 [file elife06369s003.docx]

| **MMETSP ID** | **Phylum** |  |  | **Genus** | **species** | **Strain** | **VTC2** |
| --- | --- | --- | --- | --- | --- | --- | --- |
|  |  |  |  |  |  |  |  |
| MMETSP0033 | Chlorophyta | Prasinophyceae | Mamiellaceae | Dolichomastix | tenuilepis | CCMP3274 | VTC2 |
| MMETSP0034 | Chlorophyta | Prasinophyceae | Pycnococcaceae | Nephroselmis | pyriformis | CCMP717 | VTC2 |
| MMETSP0052 | Chlorophyta | Chlorophyceae | Chlamydomonadaceae | Polytomella | parva | SAG 63-3 | VTC2 |
| MMETSP0058 | Chlorophyta | Prasinophyceae | Halosphaeraceae | Pyramimonas | parkeae | CCMP726 | VTC2 |
| MMETSP0059 | Chlorophyta | Prasinophyceae | Halosphaeraceae | Pyramimonas | parkeae | CCMP726 | VTC2 |
| MMETSP0419 | Chlorophyta | Prasinophyceae | Chlorodendraceae | Tetraselmis | sp. | GSL018 | VTC2 |
| MMETSP0491 | Chlorophyta | Prasinophyceae | Chlorodendraceae | Tetraselmis | chuii | PLY429 | VTC2 |
| MMETSP0802 | Chlorophyta | Prasinophyceae | Mamiellaceae | Micromonas | sp. | CCMP2099 | VTC2 |
| MMETSP0803 | Chlorophyta | Prasinophyceae | Unknown | Crustomastix | stigmata | CCMP3273 | VTC2 |
| MMETSP0804 | Chlorophyta | Prasinophyceae | Chlorodendraceae | Tetraselmis | astigmatica | CCMP880 | VTC2 |
| MMETSP0807 | Chlorophyta | Unknown | Unknown | Picocystis | salinarum | CCMP1897 | VTC2 |
| MMETSP0817 | Chlorophyta | Prasinophyceae | Chlorodendraceae | Tetraselmis | striata | LANL1001 | VTC2 |
| MMETSP0818 | Chlorophyta | Prasinophyceae | Chlorodendraceae | Tetraselmis | striata | LANL1001 | VTC2 |
| MMETSP0819 | Chlorophyta | Prasinophyceae | Chlorodendraceae | Tetraselmis | striata | LANL1001 | VTC2 |
| MMETSP0820 | Chlorophyta | Prasinophyceae | Chlorodendraceae | Tetraselmis | striata | LANL1001 | VTC2 |
| MMETSP0926 | Chlorophyta | Prasinophyceae | Mamiellaceae | Ostreococcus | mediterraneus | RCC789 | VTC2 |
| MMETSP0927 | Chlorophyta | Prasinophyceae | Mamiellaceae | Ostreococcus | mediterraneus | BCC10000 | VTC2 |
| MMETSP0928 | Chlorophyta | Prasinophyceae | Mamiellaceae | Ostreococcus | mediterraneus | BCC26000 | VTC2 |
| MMETSP0929 | Chlorophyta | Prasinophyceae | Mamiellaceae | Ostreococcus | mediterraneus | BCC35000 | VTC2 |
| MMETSP0930 | Chlorophyta | Prasinophyceae | Mamiellaceae | Ostreococcus | mediterraneus | BCC44000 | VTC2 |
| MMETSP0931 | Chlorophyta | Prasinophyceae | Mamiellaceae | Ostreococcus | mediterraneus | BCC49000 | VTC2 |
| MMETSP0933 | Chlorophyta | Prasinophyceae | Mamiellaceae | Ostreococcus | mediterraneus | BCC99000 | VTC2 |
| MMETSP0934 | Chlorophyta | Prasinophyceae | Mamiellaceae | Ostreococcus | mediterraneus | BCC102000 | VTC2 |
| MMETSP0935 | Chlorophyta | Prasinophyceae | Mamiellaceae | Ostreococcus | mediterraneus | BCC103000 | VTC2 |
| MMETSP0936 | Chlorophyta | Prasinophyceae | Mamiellaceae | Ostreococcus | mediterraneus | BCC109000 | VTC2 |
| MMETSP0937 | Chlorophyta | Prasinophyceae | Mamiellaceae | Ostreococcus | mediterraneus | BCC115000 | VTC2 |
| MMETSP0938 | Chlorophyta | Prasinophyceae | Mamiellaceae | Ostreococcus | mediterraneus | BCC116000 | VTC2 |
| MMETSP0939 | Chlorophyta | Prasinophyceae | Mamiellaceae | Ostreococcus | mediterraneus | BCC118000 | VTC2 |
| MMETSP1080 | Chlorophyta | Prasinophyceae | Mamiellaceae | Micromonas | sp | CCMP1646 | VTC2 |
| MMETSP1159 | Chlorophyta | Unknown | Unknown | Picocystis | salinarum | CCMP1897 | VTC2 |
| MMETSP1161 | Chlorophyta | Trebouxiophyceae | Chlorellales | Picochlorum | oklahomensis | CCMP2329 | VTC2 |
| MMETSP1169 | Chlorophyta | Prasinophyceae | Pyramimonadaceae | Pyramimonas | obovata | CCMP722 | VTC2 |
| MMETSP1315 | Chlorophyta | Prasinophyceae | Pycnococcaceae | Prasinoderma | singularis | RCC927 | VTC2 |
| MMETSP1316 | Chlorophyta | Prasinophyceae | Pycnococcaceae | Pycnococcus | provasolii | RCC2336 | VTC2 |
| MMETSP1327 | Chlorophyta | Mamiellophyceae | Mamiellaceae | Micromonas | pusilla | RCC2306 | VTC2 |
| MMETSP1330 | Chlorophyta | Trebouxiophyceae | Trebouxiophyceae | Picochlorum | sp. | RCC944 | VTC2 |

**Supplementary File 3: Identification of *VTC2* in Marine Microbial Eukaryote Transcriptome database**
