# Supplementary material for: Evolution of alternative biosynthetic pathways for vitamin C following plastid acquisition in photosynthetic eukaryotes
Source: eLife. 2015 Mar 13;4:e06369. doi: 10.7554/eLife.06369 (PMC4396506; doi:10.7554/eLife.06369)
Supplement: Supplementary file 4. — Distribution of ascorbate biosynthetic genes in Archaeplastida transcriptomes. Rhodophyte transcriptomes from the Marine Microbial Eukaryote Transcriptome Sequencing Project (MMETSP) or Genbank (Chan et al., 2011, 2012) were examined for the presence of ascorbate biosynthesis genes. The rhodophytes transcriptomes all exhibit the pathway found in the genomes of Cyanidioschyzon merolae, Chondrus crispus and Porphyridium purpureum, possessing GLDH rather than GULO. C. atmophyticus is a green alga belonging to the Streptophyte lineage containing land plants and charophyte algae. The Chlorokybus transcriptome appears unique amongst the Viridiplantate in that it contains GULO rather than GLDH. All of the other enzymes of the plant pathway are present. DOI: http://dx.doi.org/10.7554/eLife.06369.018 [file elife06369s004.docx]

|  |  |  |  |  | | | | |  | | | |
| --- | --- | --- | --- | --- | --- | --- | --- | --- | --- | --- | --- | --- |
|  |  |  |  | PLANT PATHWAY | | | | | | | ANIMAL PATHWAY | |
|  |  |  |  | VTC3 | GME | VTC2 | VTC4 | L-galdh | | GLDH | SMP30 | GULO |
| GenBank ESTs | Bangiophyceae | *Porphyra* | *umbilicalis* | *✓* | *✓* |  |  | *✓* | | *✓* |  |  |
| GenBank ESTs | Bangiophyceae | *Porphyra* | *purpurea* | *✓* | *✓* |  |  | *✓* | | *✓* |  |  |
|  |  |  |  |  |  |  |  |  | |  |  |  |
| MMETSP0312 | Compsopogonophyceae | *Compsopogon* | *coeruleus* | *✓* | *✓* |  |  | *✓* | | *✓* |  |  |
| MMETSP0011_2 | Rhodellophyceae | *Rhodosorus* | *marinus* | *✓* | *✓* |  | *✓* | *✓* | | *✓* |  |  |
| MMETSP0315 | Rhodellophyceae | *Rhodosorus* | *marinus* |  | *✓* |  | *✓* | *✓* | | *✓* |  |  |
| MMETSP0167 | Rhodellophyceae | *Rhodella* | *maculata* |  | *✓* |  |  | *✓* | | *✓* |  |  |
| MMETSP0314 | Rhodellophyceae | *Rhodella* | *maculata* |  | *✓* |  |  | *✓* | | *✓* |  |  |
| MMETSP0313 | Porphyridiophyceae | *Porphyridium* | *aerugineum* |  | *✓* |  |  | *✓* | | *✓* |  |  |
| MMETSP1172 | Porphyridiophyceae | *Timspurckia* | *oligopyrenoides* |  |  |  |  |  | | *✓* |  |  |
| MMETSP1353 | Porphyridiophyceae | *Erythrolobus* | *australicus* |  | *✓* |  |  |  | | *✓* |  |  |
|  |  |  |  |  |  |  |  |  | |  |  |  |
| GenBank TSA | Chlorokybophyceae | *Chlorokybus* | *atmophyticus* | *✓* | *✓* | *✓* | *✓* | *✓* | |  | *✓* | *✓* |

**Supplementary File 4: Distribution of ascorbate biosynthetic genes in selected Archaeplastida transcriptomes**
