# Supplementary material for: Evolution of alternative biosynthetic pathways for vitamin C following plastid acquisition in photosynthetic eukaryotes
Source: eLife. 2015 Mar 13;4:e06369. doi: 10.7554/eLife.06369 (PMC4396506; doi:10.7554/eLife.06369)
Supplement: Supplementary file 5. — Genome resources used in this study. A list of the eukaryote genomes used to study the distribution of genes relating to ascorbate biosynthesis and metabolism. DOI: http://dx.doi.org/10.7554/eLife.06369.019 [file elife06369s005.docx]

| **Taxon ID** | **Supergroup** | **Species** | **Genome source** | **Genome**  **version** |
| --- | --- | --- | --- | --- |
| 3702 | Archaeplastida | *Arabidopsis thaliana* | http://phytozome.jgi.doe.gov | TAIR10 |
| 39947 |  | *Oryza sativa* | http://phytozome.jgi.doe.gov | v7 |
| 4577 |  | *Zea mays* | http://phytozome.jgi.doe.gov | v6a |
| 88036 |  | *Selaginella moellendorffii* | http://phytozome.jgi.doe.gov | v1.0 |
| 3218 |  | *Physcomitrella patens* | http://phytozome.jgi.doe.gov | v1.6 |
| 3055 |  | *Chlamydomonas reinhardtii* | http://phytozome.jgi.doe.gov | v5.3.1 |
| 3068 |  | *Volvox carteri* | http://phytozome.jgi.doe.gov | v2.0 |
| 554065 |  | *Chlorella variabilis* | http://genome.jgi-psf.org/ChlNC64A_1/ChlNC64A_1.home.html | v1.0 |
| 70448 |  | *Ostreococcus tauri* | http://genome.jgi-psf.org/Ostta4/Ostta4.home.html | v2.0 |
| 242159 |  | *Ostreococcus lucimarinus* | http://phytozome.jgi.doe.gov | v2.0 |
| 296587 |  | *Micromonas RCC 299* | http://phytozome.jgi.doe.gov | v3.0 |
| 2769 |  | *Chondrus crispus* | http://www.ncbi.nlm.nih.gov/genome/12106 |  |
| 45157 |  | *Cyanidioschyzon merolae* | http://merolae.biol.s.u-tokyo.ac.jp/t |  |
| 130081 |  | *Galdieria sulphuraria* | http://www.ncbi.nlm.nih.gov/genome/405 |  |
| 1389228 |  | *Galdieria phlegrea* | http://cyanophora.rutgers.edu/gphlegrea/ |  |
| 2762 |  | *Cyanophora paradoxa* | http://cyanophora.rutgers.edu/cyanophora/home.php |  |
|  |  |  |  |  |
| 67593 | Stramenopiles | *Phytophthora sojae* | http://genome.jgi-psf.org/Physo3/Physo3.home.html | v3.0 |
| 296543 |  | *Thalassiosira pseudonana* | http://www.ncbi.nlm.nih.gov/genome/54 |  |
| 556484 |  | *Phaeodactylum tricornutum* | http://www.ncbi.nlm.nih.gov/genome/418 |  |
| 186039 |  | *Fragilariopsis cylindrus* | http://genome.jgi-psf.org/Fracy1/Fracy1.home.html | v1.0 |
| 2880 |  | *Ectocarpus siliculosus* | http://www.ncbi.nlm.nih.gov/genome/2704 |  |
| 5888 | Alveolates | *Paramecium tetraurelia* | http://paramecium.cgm.cnrs-gif.fr/ |  |
| 5911 |  | *Tetrahymena thermophila* | http://ciliate.org/index.php/home/welcome |  |
| 753081 | Rhizaria | *Bigelowiella natans* | http://genome.jgi.doe.gov/Bigna1/Bigna1.home.html | v1.0 |
| 46433 |  | *Reticulomyxa filosa* | http://www.ncbi.nlm.nih.gov/genome/11245 |  |
|  |  |  |  |  |
| 280463 | CCTH | *Emiliania huxleyi* | <http://genome.jgi.doe.gov/Emihu1/Emihu1.home.html> | v1.0 |
| 905079 |  | *Guillardia theta* | http://genome.jgi.doe.gov/Guith1/Guith1.home.html | v1.0 |
|  |  |  |  |  |
| 5691 | Excavata | *Trypanosoma brucei* | http://www.ncbi.nlm.nih.gov/genome/24 |  |
| 3039 |  | *Euglena gracilis** | *sequenced transcriptome* |  |
| 5762 |  | *Naegleria gruberi* | http://www.ncbi.nlm.nih.gov/genome/264 | v1.0 |
|  |  |  |  |  |
| 44689 | Amoebozoa | *Dictyostelium discoideum* | http://www.ncbi.nlm.nih.gov/genome/56 |  |
|  |  | *Acanthamoeba castellanii* | http://www.ncbi.nlm.nih.gov/genome/278 |  |
|  |  |  |  |  |
| 9606 | Opisthokonts | *Homo sapiens* | http://www.ncbi.nlm.nih.gov/genome/51 |  |
| 10090 |  | *Mus musculus* | http://www.ncbi.nlm.nih.gov/genome/52 |  |
| 7227 |  | *Drosophila melanogaster* | http://www.ncbi.nlm.nih.gov/genome/47 |  |
| 10228 |  | *Trichoplax adhaerens* | http://www.ncbi.nlm.nih.gov/genome/354 | v1.0 |
| 81824 |  | *Monosiga brevicollis* | http://www.ncbi.nlm.nih.gov/genome/713 | v1.0 |
| 946362 |  | *Salpingoeca rosetta* | http://www.broadinstitute.org/ |  |
| 192875 |  | *Capsaspora owczarzaki* | http://www.broadinstitute.org/ | v1.0 |
| 4932 |  | *Saccharomyces cerevisiae* | http://www.yeastgenome.org/ |  |
| 1123529 |  | *Gonapodya prolifera* | http://genome.jgi.doe.gov/Ganpr1/Ganpr1.home.html | v1.0 |
| 529818 |  | *Thecamonas trahens* | http://www.broadinstitute.org/ |  |

**Supplementary File 5: Assembled genomes used in this study**
